# Spirituality and the Use of Psychoactive Substances: The Perspective of Polish Students 

**DOI:** 10.1007/s10943-023-01748-0

**Published:** 2023-02-03

**Authors:** Marzena Chrost, Sławomir Chrost

**Affiliations:** 1 Jesuit University Ignatianum in Krakow, ul. Kopernika 26, 31-501 Kraków, Poland; 2 Jan Kochanowski University in Kielce, ul. Krakowska 11, 25-029 Kielce, Poland

**Keywords:** Spirituality, Protective factors, Students, The use of psychoactive substances, Poland

## Abstract

The objective of the article is to examine and analyze the correlation between the spirituality of Polish students and their use of psychoactive substances. The main research problem has been formulated as follows: *What is the relationship between students’ spirituality and their use of selected psychoactive substances?* The empirical research used the diagnostics survey method with the questionnaire technique and ASP (Aspects of Spirituality) scales. The study was carried out from November 2021 to December of the same year. The participants included 1348 Polish students from Krakow academies and universities. Based on the results of statistical tests, the hypothesis on the correlation between the students’ spirituality and their use of selected psychoactive substances was verified and accepted. Students with higher levels of spirituality (the protective factor) use psychoactive substances less frequently and in smaller quantities. Therefore, it was concluded that spirituality is a protective factor in young peoples’ lives. Some implications for student education are noted.

## Introduction

In his work, *Megatrends in Postmodern Societies,* Janusz Mariański, a well-known and respected Polish sociologist, describes global phenomena that influence the main changes taking place in the world. Although these phenomena are taking shape slowly, they are affecting many areas of human life. Apart from secularization, desecularization, social and cultural pluralism, individualization and globalization, Mariański indicates new spirituality as a social and cultural megatrend (Mariański, 2016).

Another trend, or perhaps megatrend, is the use of psychoactive substances. This is nothing new, but in times of social and cultural changes, chaos, or even lockdowns, it seems that these substances are being used more frequently and in larger quantities. According to a study that was carried out before the pandemic, drug use is very popular among young people. As many as 90.91% of the girls and 87.23% of the boys who took part in the study admitted that they used psychoactive substances (Szpringer et al., 2015). The United Nations Office on Drugs and Crime (UNODC, 2020) also reports an increase in drug use and a significant influence of the COVID-19 pandemic on drug markets all over the world (UNODC, 2020).

Studies focus on the period in which adolescents and young adults enter a new social group. Moreover, we can observe that today’s students often pursue two or more majors at the same time, and most of them are working or participating in internships. They live a hectic life, which is marked by impatience and lack of time resulting from taking up many different social roles. For this reason, this social group is particularly exposed to negative behavior patterns and to psychoactive substances. It has to be emphasized that any possible use of psychoactive substances may create serious risks of health (physical and mental) as well as of social harm, and can significantly influence students’ adult life. Therefore, more effective actions should be taken to promote a healthy lifestyle. For the sake of the wellbeing of students and, in the long run, the wellbeing of the whole society, it is necessary to monitor the scale of psychoactive substance abuse in order to organize preventive measures aimed at limiting this problem and reducing the risk of addiction.

The objective of this paper is to present a study on the correlation between students’ spirituality and their use of selected psychoactive substances. First, we will show the basic theoretical concepts related to spirituality and psychoactive substances. Then, we will outline the methodological principles of our empirical research and characterize the students who took part in the survey. Next, we will describe the findings related to students’ intake of psychoactive substances, and discuss the components of their spirituality in this context. In the conclusions and implications section, we will show the role of spirituality as a factor that determines the use of psychoactive substances.

## Basic Concepts

### Spirituality and “New Spirituality”

Spirituality is a concept that is theorized by various scientific disciplines. Below we will only provide its selected and exemplary definitions.

Marek Chmielewski, a theologian of spirituality, while referring to the anthropology of Karol Wojtyła, has developed a unique thesis on anthropogenic spirituality which derives from human nature. A special form of such spirituality, in the subjective aspect, is Christian spirituality. It is supernatural and cannot be reduced to any other forms of spirituality (Chmielewski, 2020).

James Wiseman distinguishes three ways of understanding the notion of spirituality. The first way defines spirituality as the ability to transcend oneself in order to achieve the ultimate value, i.e. overcoming one’s egoism and pursuing perfection (understood in a lay or religious manner). The second way is searching for unity with the surrounding world and everything that exists (e. g. with people, nature, etc.) in experiencing one’s true self. The third way of perceiving spirituality is connected with the Christian concept of a mystical connection with God achieved through “living faith” and grace that makes it possible to attain the highest perfection or aim, i.e. holiness (Wiseman, 2009).

Nowadays we can also hear about new spirituality as a phenomenon developing due to social and cultural changes taking place in the world. This is what Halina Mielicka writes about “new spirituality”: “so-called *new spirituality*, spirituality of the end of the 20th and the beginning of the twenty-first century, is perceived as people’s fight with technicization, secularization, economic and industrial globalization, media and political manipulation, cultural homogenization, and environmental disaster resulting from the destruction of nature. Especially young people are interested in spirituality, not so much at the level of ideas, but at the level of spiritual experience” (Mielicka, 2011, p. 9).

While thinking about various approaches to “new spirituality”, Janusz Mariański enumerates the following characteristic features that recur in many definitions of this phenomenon:

- New spirituality has its source in various religious and spiritual traditions;
- An important feature of what we call “new spirituality” is the focus on experience and feeling (experiencing transcendence manifests itself both in extreme acts, such as sudden conversion, and in ordinary experiences);
- Spirituality and new spirituality are connected with the process of individualization and, through their focus on subjectivity, are often in opposition to institutionalized religions (subjectivization means emphasizing the idea of development and self-development; of personal and inner self-perfection);
- Many forms of new spirituality can be defined as post-religious spirituality, or even spirituality without God;
- New spirituality is usually placed in opposition to religion, and sometimes even to the sacred (it is generally connected with a form of “little transcendence”, crossing the biological human condition, searching for values that bring meaning to life, psychological well-being, positive lifestyle, etc.);
- New spirituality cannot be identified only with new religious movements or New Age as it has become a distinctive trend or megatrend in the modern world;
- New spirituality is related to values that include spiritual development, inner improvement, the ethical or aesthetical dimension of the human “self”, a sense of happiness and fulfillment in life (important values include what constitutes good for an individual; what is connected with the search for meaning and interpretation of life);
- Religious faith transforms into faith in one’s own “I”, in subjectivity; there is a shift from the community to the individual, and the focus is on immanent values and objectives;
- New spirituality is often connected with searching for and giving meaning both to a person’s everyday experiences and to his/her entire existence (Mariański, 2016).

### Psychoactive Substances

As we have noted in the introduction, psychoactive substances are in widespread use. They are also available to young people, including students.

Zbigniew Michalczyk defines psychoactive substances as “chemical substances that activate the central nervous system, and directly influence brain functions, as a result of which people experience temporary changes in perception, mood, awareness and behavior” (Michalczyk, 2018, p. 105).

According to the classification of the UN and INTERPOL, psychoactive substances are divided into those that have depressant, stimulant and disruptive effects on the central nervous system.

Also, psychoactive substances can be classified according to the substance and its origin. The substances may be natural, semi-synthetic or synthetic. According to the legal classification of substances, there are legal and illegal drugs.

According to the classification of the Addiction Research Foundation, psychoactive substances include:

- Depressants—Alcohol, sleeping and sedative pills, and inhalants.
- Stimulants—Cocaine, and amphetamines.
- Hallucinogens—Marihuana, LSD, psilocybin, mescaline and “designer” drugs.
- Opiates—Heroin, morphine, codeine, opium, and methadone (Michalczyk, 2018).

According to the current classification of the World Health Organization, psychoactive substances are divided into six groups. One of the classification criteria is their pharmacological activity (Schuckit, 1990). Thus, we can distinguish between:

- Central nervous system depressants, i.e. substances that reduce the symptoms of activity of the central nervous system (CNS) (e.g. ethyl alcohol, barbiturates, benzodiazepines and *γ*-hydroxybutyric acid).
- CNS stimulants, i.e. psychoactive substances that stimulate the activity of CNS (such as amphetamine, methamphetamine, cocaine, methcathinone, cathine and cathinone).
- Opioids: morphine, codeine, thebaine, as well as semi-synthetic and synthetic opioids.
- Cannabinoids, i.e. cannabinoid components of Cannabis sativa, which is known on the drug market as marihuana, hashish and hash oil.
- Hallucinogens, i.e. substances that produce hallucinations, which are usually divided into four subgroups belonging to different chemical categories:

- Ergoline derivatives (lysergic acid),
- Tryptamine derivatives (psilocin and psilocybin),
- Carboline derivatives (harmine and harmaline),
- Phenethylamine derivatives (mescaline).

Inhalants or volatile substances are a large group of substances used mainly by very young people, including primary school students. Young people choose them because they are readily available, cheap and easy to use (inhale). Inhalants include common solvents, such as toluene, xylene, hexane and benzene, gases, such as propane and butane, aerosol propellants, and sprays.

### Spirituality and Psychoactive Substance Abuse

According to Tomasz Sikora, the ritual use of hallucinogens can be traced back to the ninth century B.C. The use of hallucinogens is also confirmed for Mesoamerican religions. The psychedelic revolution of 1960s was a key factor behind the development of the New Age Movement (Sikora, 1999). Sikora claims that in traditional communities collective rituals with the use of hallucinogens made it easier for people to internalize a desired pattern of behaviours and the information associated with a specific social role (Sikora, 1999).

Marek Dziewiecki notes that a person who has not developed their spirituality becomes addicted to their body, subjective thinking, emotions, pressures from the world around them, particular people, substances or things. A person devoid of spiritual life is, in a way and to some extent, addicted to all of those factors simultaneously. According to Dziewiecki, mass media in the modern world promote a lifestyle that ignores spirituality, which leads to addictions. The dominance of the man of flesh over the man of the spirit inevitably leads to an increase in the number of people addicted to various substances (Dziewiecki, 2001).

As Dziewiecki writes, “The abuse of psychoactive substances is a sign of looking for easy happiness which does not exist. It is succumbing to the illusion that we can improve our mood without changing our lives and without correcting our past behaviour. Such an attitude makes us escape from spirituality and, in consequence, from reality. Spirituality is the search for an objective, i.e. holistic and realistic perception of a person, and such a philosophy of life that leads to the optimal development of a person. An addict, on the other hand, seeks oblivion, feeds on subjective illusions, compulsively deceives themselves, and follows a destructive philosophy of life, i.e. strives to improve their mood in a way other than by modifying their behaviour and situation in life” (Dziewiecki, 2006, p. 5).

Beata Zajęcka carried out research in order to identify the role of spirituality in the treatment of people addicted to psychoactive substances. Her research showed that an addict’s spirituality becomes degraded and destroyed by psychoactive drugs. An addict does not know who they are and why they live; they cannot direct their lives freely and consciously. As their spirituality is not shaped, they cannot be truly free. Thus, according to Zajęcka, it is important to develop spirituality from the very beginning, in parallel with other therapeutic activities (Zajęcka, 2016).

The positive role of spirituality as a factor that protects or supports alcohol addicts has been confirmed by another study. According to a study carried out using the individual case method (the technique of the individual in-depth interview), among 10 alcohol addicts (6 men and 4 women), who decided to undergo therapy and abstained from alcohol for at least a year, the role of spirituality in the therapeutic process is significant. Spirituality helps people abstain from drinking, because an alcoholic who feels the support of a higher being is motivated to act (Chrost, 2020).

At present, it can be seen that people are increasingly interested in spirituality in its various forms and types. Also, one of the symptoms of the modern world is spiritual emptiness, which is manifests itself mainly in a sense of purposelessness and meaninglessness of life or in limiting the meaning and purpose of life to a short-term, hedonist perspective. Another alarming sign of our times is that many people find financial prosperity more important than spiritual development. At the same time, more and more people are turning to psychoactive substances. This trend peaks in early adulthood. According to research, this problem also affects a significant group of students (Pach et al., 2005; Kupiec, 2012; Rogowska, 2015; Karczewska et al., 2016; Gacek et al., 2021). However, in books discussing this issue, there are still few research papers that describe the scale of such substance abuse among Polish students (Rogowska, 2015). This has become an inspiration for us to carry out our empirical research aimed at examining and analyzing the correlation between students’ spirituality and their substance abuse in Poland.

### Research Methods

The main research problem was defined as follows: *What is the relationship between students’ spirituality and their use of selected psychoactive substances?* The following hypothesis was formulated with regard to the problem: It is assumed that there is a correlation between students’ spirituality and their use of selected psychoactive substances. The higher the level of spirituality (a protective factor), the lower the frequency and quantity of drug use by young people.

In order to answer the above question and verify the hypothesis, the authors of the study applied the diagnostic survey method with the questionnaire technique. Also, the ASP (Aspects of Spirituality) questionnaire was used^1^ (Büssing et al., 2007, 2014). This questionnaire can measure a broad range of important aspects of spirituality. It takes into account both religious and secular forms of spirituality. This tool avoids exclusionary language (the word “God” was only used once and can be easily replaced with other terms in different cultural contexts) and situates informal aspects of spirituality in terms of relational awareness. It can be used for studying spirituality of both religious people and people with sceptical or non-religious attitudes.

The ASP questionnaire distinguishes four factors of spirituality:

- Religious orientation (alpha = 0.93), i.e. prayer; guidance and refuge; trusting and seeking help from God; spiritual orientation in life; separate rituals; reading spiritual/religious books, etc.
- Seeking insight/wisdom (alpha = 0.88), i.e. knowledge and truth; development of wisdom; beauty/goodness; honesty/ deep spirituality; broad awareness, etc.
- Conscious interactions (alpha = 0.83), i.e. conscious interactions with others, with other people, with oneself, empathy and generosity.
- Belief in transcendence (alpha = 0.85), i.e. the existence of higher beings; in the rebirth of a person/soul; in the soul originating from higher planes; and in a person as a spiritual creature (Büssing et al., 2014).

In the questionnaire, the authors included questions referring to only six selected psychoactive substances that is alcohol, nicotine, marihuana, energy drinks, designer drugs, and amphetamines. This was because these are the most popular psychoactive substances used by Polish students. The students who took part in the study were asked how often they take these substances and in what quantities. A five-point answer scale was prepared for the questions on frequency (very often, often, sometimes, rarely, never), and a six-point scale was prepared for the questions on quantity (very large, large, moderate, small, very small, none). Moreover, it was possible for the students to mention other psychoactive substances (not listed in the questionnaire) and to specify the frequency and quantity of their intake.

The research was carried out from November 2021 to December of the same year. Contacting the students directly was not possible because of the restrictions resulting from the COVID-19 pandemic. Therefore, the safest and the most effective form of collecting research material were to create an online survey questionnaire which was made available on the Survio platform.^2^ The authors of the study obtained the approval of proper authorities for carrying out the survey in selected Krakow universities. The study obtained the approval and consent of the university Commission for Scientific Research Ethics, and was carried out in accordance with all national and international ethical standards.

Information about the research was sent to the students through university emails. The request to fill in the questionnaire was preceded with a letter explaining the purpose and scope of the research, along with a link to the survey. Participation in the survey was anonymous and voluntary, and participants were given the opportunity to opt out of completing and sending the questionnaire. It is worth noting that 2975 students opened the link with the survey questionnaire, and only 1370 of them filled it in, which means that 1605 students failed to complete it (the completion rate was less than half, i.e. 46.10%). This may indicate that the problem in question refers to difficult, personal, intimate and sensitive issues. On the other hand, there can be other, unknown reasons why the students did not want to answer those questions. This interesting issue can be addressed in other, more detailed studies.

After verification and selection of the collected data, 1348 properly completed questionnaires were finally qualified for a statistical quantitative analysis.

Statistical analyses were carried out using the statistical package PQStat, version 1.8.2.230. The statistical analysis was done with the use of the descriptive statistical methods and properly selected tests. The connection between gender and the frequency of using particular substances was analyzed with the Pearson’s (*χ*2) chi-squared test and Fisher’s exact test. In order to find out the relationship between spirituality scales and the quantity of psychoactive substances taken, cluster analysis using k-means was carried out. The test probability of *p* < 0.05 was considered to be significant, and the probability of *p* < 0.01 was deemed highly significant.

### Characteristics of the Students Who Took Part in the Study

Krakow is very often referred to as the capital city of Polish student life. There are more than a dozen large academies and universities in the city. Each of them educates several thousand people at many different faculties. The request for filling in the questionnaire was directed to people who attend full-time studies in Krakow. A total number of 1348 students took part in the research, including 65.30% women (880 respondents) and 34.70% men (468 respondents). Thus, significantly more women than men participated in the survey. The reason for this is that more women than men take up studies in Poland.

The students are adults who generally start university education at the age of 18–19 or more. The students surveyed were between the ages of 18 and 40. The largest group (62.24%) was people aged 18–21 (839 respondents). The average age of the students was *M* = 21.36, and the standard deviation was SD = 2.57.

The students were from different Krakow academies and universities: 40.43% studied at the Jagiellonian University (545 respondents), 25.07% studied at Hugo Kołłątaj University of Agriculture (338 respondents), 16.62% were students of the AGH University of Science and Technology (224 respondents), and 15.43% were students of the Jesuit University Ignatianum (208 respondents). The remaining 2.45% (33 respondents) were a small number of students of other Krakow universities, such as the University of Economics, the Pontifical University of John Paul II, Tadeusz Kościuszko University of Technology, and Tischner European University (see Table 1).

**Table 1:** Baseline characteristics of respondents

Variable	Gender		Age			
	Women	Men	18–21	22–25	26–29	30–40
Number (*N*)	880	468	839	443	42	24

Name of the university	Number (*N*)
Jagiellonian University	545
Hugo Kołłątaj University of Agriculture	338
AGH University of Science and Technology	224
Jesuit University Ignatianum	208
Other Krakow universities	33

## Result Analysis

### Use of Psychoactive Substances by Students

During studies, a young person develops and discovers themselves and builds their identity. It is a time of becoming an adult, learning, competing, obtaining new intellectual and professional competences, as well as experimenting with the use of psychoactive substances. Therefore, the students who took part in the survey were asked to specify the frequency of use of six selected psychoactive substances that is alcohol, nicotine, marihuana, energy drinks, designer drugs, and amphetamines. The students could also mention other substances (not named in the questionnaire), but this data were not included or discussed in this article because of the scarcity of such answers.

Analysis of the data given by the students shows that alcohol is the most popular psychoactive substance in this group. As many as 92.88% of the students (1252 respondents) said that they drink alcohol. This is undoubtedly related to its low price and availability, as the sales network of alcoholic beverages is very extensive. Moreover, “alcohol is part of Polish culture and tradition. Drinking alcohol is associated with family celebrations and church feasts. It accompanies us at home and at work; it is connected with birth and death. Growing up in this cultural and traditional context, we learn and adopt certain ways and attributes of celebrating important events in our lives” (Michalczyk, 2018, p. 78).

In the group of students under study only 7.12% (96 respondents) said that they had never drunk alcohol. This is alarming, because alcohol is the third most dangerous risk factor for the health of the general population. It causes a lot of diseases and disorders, results in premature deaths and increases death rates in society (WHO).

The second most popular psychoactive substance among students is energy drinks. More than two third of students (70.18%—946 respondents) admitted that they use such drinks. More than one fourth, or 29.82% of students (402 respondents) declared that they had never drunk energy drinks. The third psychoactive substance used by the students was nicotine, as more than half of the respondents (51.56%) have experience smoking cigarettes (695 respondents). Almost half of the students (48.44%—653 respondents) said that they had never smoked. Marihuana is used by 37.24% of the students (502 respondents), while 62.76% of them (846 respondents) admitted that they had never used that substance.

Amphetamines are used by 5.26% students (71 respondents); and 94.74% (1277 respondents) have never taken it. We may assume that the low rate of amphetamine users may result from the fact that only few people admit taking it, even in anonymous surveys, since taking drugs is socially unacceptable.

The smallest number of students, i.e. only 3.26% (44 respondents) reported that they had taken designer drugs, and 96.74% students (1304 respondents) declared that they had never taken them. It is worth mentioning that designer drugs and inhalants are toxic and may cause irreversible damage in the body even after a single use, which should definitely prompt the authorities to take preventive measures to reduce the number of young people taking these substances.

The students who took part in the survey were also asked to specify how often they took psychoactive substances. The details are shown in Table 2.

**Table 2:** The frequency of use of selected psychoactive substances by the students

Frequency of use	Alcohol		Nicotine		Marihuana		Energy drinks		Designer drugs		Amphetamine	
	L	%	L	%	L	%	L	%	L	%	L	%
Very often (at least twice a week)	153	11.35	287	21.29	37	2.74	134	9.94	6	0.44	7	0.52
Often (e.g. once a week)	384	28.49	65	4.82	40	2.97	174	12.91	2	0.15	4	0.29
Sometimes (e.g. once a month)	552	40.95	139	10.32	134	9.94	329	24.41	12	0.89	20	1.48
Rarely (e.g. once a year)	163	12,09	204	15.13	291	21.59	309	22.92	24	1.78	40	2.97
Never	96	7.12	653	48.44	846	62.76	402	29.82	1304	96.74	1277	94.74
Total	1348	100	1348	100	1348	100	1348	100	1348	100	1348	100

According to the data analysis, the frequency of use of psychoactive substances by students is varied. Only the highest percentages in the frequency and quantity of use will be discussed in detail. The largest number of students (40,95%, i.e. 552 respondents) said that they sometimes (once a month) drink alcohol. As for the quantity, the largest number of respondents (45.85% students, i.e. 618 respondents) said that they use moderate amounts. Almost one fourth of the students (24.41%—329 respondents) sometimes (once a month) use energy drinks, and more than a quarter of them (219 respondents), 25.22%, reported that they use small quantities of such drinks. Marihuana is rarely (once a year) used by 21.59% of the students (291 respondents). The largest group, i.e. 19.65% of the people who took part in the study (231 respondents), use very small quantities of marihuana. Thus, marihuana is still a popular psychoactive substance among the students. We may suspect that its calming and soothing influence helps young people relax before the exams (cf. Gacek, 2021). More than one fifth of the students, i.e. 21.29%, (287 respondents) said that they use nicotine very often (at least twice a week). The highest percentage of them (17.14%) declared that they use very small quantities of nicotine. Amphetamine is used by 2.97% students (40 people) rarely (once a year); for 4.45% students (60 respondents) these are very small quantities. Designer drugs are also rarely used by students: 1.78% of them (24 respondents) use them in very small quantities (this was declared by 3.86% of those who took part in the research).

It is also important and interesting to analyze differences in the use of psychoactive substances by students depending on sex. Such analysis was carried out with the help of Pearson’s (*χ*2) chi-squared test and Fisher’s exact test. The outcomes show that the relationship between substance use and gender is insignificant for nicotine and energy drinks (*p* > 0.05). However, for other psychoactive substances, such as alcohol, marihuana, amphetamine, and designer drugs, the correlation between the frequency of use and gender is highly significant.

As can be seen from the statistical analysis (Pearson’s chi-square *χ*2 = 31.4128; degrees of freedom d*f* = 4; *p* ≤ 0.001; Cochran’s condition was met and Fisher’s exact test *p* < 0.0001), the relationship between gender and frequency of alcohol use is highly significant and men use it more frequently than women. Here, we have to point out that, in general, in Europe sex gender influences the style of drinking alcohol, as female students drink less alcohol and use alcohol less frequently than male students.

It is also worth noting that “the limits of social acceptance for psychoactive substance use have been extended, and this does not only apply to alcohol drinking or smoking cigarettes. For example, marihuana is generally accepted by young people although it is an illegal and addictive substance. The situation is different with designer drugs, which most young people consider to be legal” (Michalczyk, 2018, p. 88). The most popular psychoactive substances among students are marihuana and amphetamines. Therefore, the data on the frequency of using these substances were analyzed according to the sex of the students.

Based on the statistical analysis (Pearson’s chi-square *χ*2 = 28.531; degrees of freedom d*f* = 4; *p* ≤ 0.001; Cochran’s condition was met, and Fisher’s exact test *p* < 0.0001), we may conclude that the relationship between gender and frequency of using marihuana is significant and men use it more frequently. The statistical analysis (Pearson’s chi-square *χ*2 = 25.8353; degrees of freedom d*f* = 4; *p* ≤ 0.001; Cochran’s condition was not met, and Fisher’s exact test *p* < 0.0001), also shows that the association between gender and frequency of using amphetamines is significant and men use this drug more frequently.

Use of designer drugs is also influenced by the sex of the students who took part in the study. According to the statistical analysis (Pearson’s chi-square *χ*2 = 10.5629; degrees of freedom d*f* = 4; *p* ≤ 0.0319; Cochran’s condition was not met, and Fisher’s exact test *p* < 0.0341), the relationship between gender and frequency of using designer drugs is significant (*p* < 0.05) and men use them more frequently.

To sum up, it is worth mentioning that in the analyzed group of students men use alcohol, marihuana, amphetamines, and designer drugs more frequently than women. We may assume that women are more stress-resistant and deal with unfamiliar situations better than men who use such substances to release stress or forget about their problems, failures, loneliness, and stressful situations. Men may also take drugs to accelerate the pace of life or just to relax. It seems that, apart from psychological aspects, the fact that male students use psychoactive substances more frequently than female students results from social and historical factors. In the Polish tradition, men used to drink alcohol at gatherings, while women who drank faced social disapproval.

It has to be admitted that the use of psychoactive substances is a serious health problem resulting in various behavioral and psychological disorders. We have not analyzed in detail the reasons for taking such substances, but we can assume that one of the factors that facilitates using them are people’s expectations. Students probably expect some positive results and willingness to gain new experiences. Using such substances may also be a form of escaping from problems, searching for oneself, and result from thinking that it will help one to overcome stress and tension in order to cope better with everyday life. Also, using alcohol and drugs may be directly connected with experiencing personal problems or with the pressure of the peer group (cf. Gacek, 2021).

### Components of Students’ Spirituality in Relation to the Use of Selected Psychoactive Substances

The ASP (Aspects of Spirituality) questionnaire was used in order to learn about the components of the spirituality of the students who took part in the research. (Büssing et al., 2007, 2014). Data analysis was carried out by calculating the mean, median and standard deviation.

Based on the data analysis, the highest scores on the ASP scale were for Searching for insight/wisdom, as its arithmetic mean was *M* = 77.7; median: Me = 78.6; and standard deviation SD = 16.3. Thus, it can be concluded that in the studied group of students most people represent this aspect of spirituality. The students seek insight and truth, developing wisdom, beauty/goodness, honesty/deep spirituality, and broad awareness. This is consistent with the theory presented at the beginning of the essay. The findings confirm the modern trend for developing Gnostic spirituality among young people (especially students) who are seeking truth and wisdom. The results on the Conscious interactions scale were slightly lower: the arithmetic mean was *M* = 73.4; median: Me = 75.0; and the standard deviation was SD = 15.9. Based on this, we can conclude that the students are curious about interactions with others, with the community, with one another; they are friendly and generous. On the Belief in transcendence scale, the mean was *M* = 58.2, median: Me = 62.5; and the standard deviation was SD = 30.4. This indicates that the students believe in the existence of higher beings, in the rebirth of a human being/soul, in the soul’s origin in higher planes, and in the idea that a person is a spiritual creature. The lowest scores with the mean *M* = 44.0, median Me = 44.4, and standard deviation DS = 31.5, were noted for Religious orientation, which refers to prayer, guidance and protection, trusting and turning to God, spiritual orientation in life, separate rituals, and reading spiritual/religious books. These findings also confirm some general trends related to a shift away from institutionalized religious forms and practices and towards subjective feelings/experiences and individualized expressions of spirituality.

**Fig. 1:**
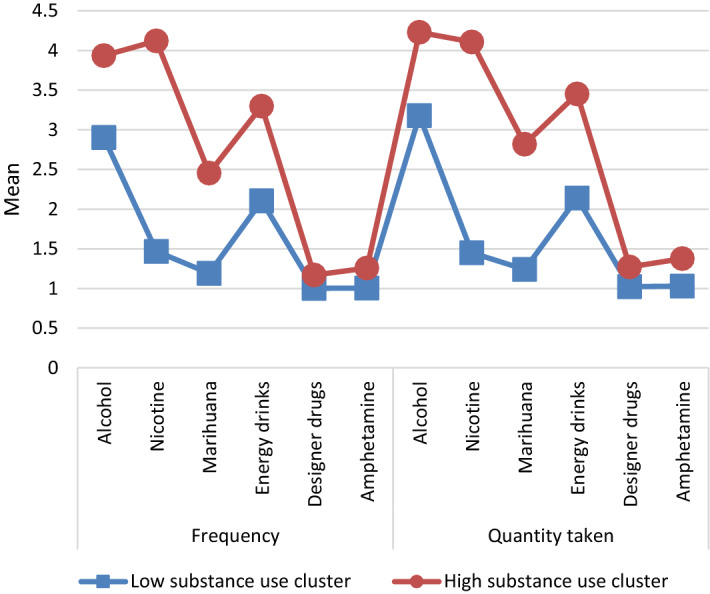
Division into two clusters using the *k*-mean method

A k-means cluster analysis was used to explore the relationship between those four spirituality scales and the frequency/quantity of use of particular psychoactive substances. The group of students under study was divided into two distinct clusters. The first cluster included those who take psychoactive substances less frequently and in smaller quantities. The other cluster included those who declare using psychoactive substances more frequently and in larger quantities. The detailed data are shown in Fig. 1.

According to the analysis of the data, the clusters are very clear and unambiguous. Next, the relationship of those clusters with students’ spirituality was studied using the t-Student test. The detailed results are shown in the Table 3.

**Table 3:** ASP spirituality scales according to clusters

		ASP religious orientation		ASP—searching for insight/wisdom		ASP—conscious interactions		ASP—belief in transcendence	
		Cluster 1	Cluster 2	Cluster 1	Cluster 2	Cluster 1	Cluster 2	Cluster 1	Cluster 2
Arithmetic mean		49.1	33.7	77.8	77.3	74.1	72.2	60.2	54.2
Mean standard error		1.1	1.3	0.5	0.8	0.5	0.8	1.0	1.4
Standard deviation		32.2	27.4	15.9	17.2	15.3	16.9	30.5	29.8
Mean difference		15.3993		0.4864		1.8424		6.0505	
− 95% CI for the mean difference		12.0988		− 1.4206		− 0.0189		2.6211	
+ 95% CI for the mean difference		18.6999		2.3935		3.7037		9.48	
Difference standard error		1.682		0.9716		0.9483		1.7482	
T-student test	*t*	9.1552		0.5006		1.9429		3.4611	
	d*f*	1036.9713		833.367		823.61		1346	
	*p*	< 0.0001		0.6168		0.0524		0.0006	

The *t* test revealed significant differences *t*(1036) = 9.1552; *p* < 0.0001. Thus, we can see a significant (*p* < 0.01) difference between the Religious orientation scale in the cluster of students who use fewer amounts of psychoactive substances (Cluster 1) and the same scale in the cluster of students who use more such substances (Cluster 2). Similarly, the t test revealed the differences *t*(1346) = 3.4611; *p* = 0.0006 in the Belief in transcendence scale. There is a highly significant difference (*p* < 0.01), i.e. the cluster that includes students who do not use a lot of psychoactive substances (Cluster 1) has a higher mean in the ASP scale: Belief in transcendence than the mean of the cluster in which people use more psychoactive substances (Cluster 2). In case of the ASP scales: Searching for Insight/wisdom; Conscious interactions, there were no significant differences (*p* > 0.05) between those two clusters.

### Research Limitations

The outcomes of the study should be viewed in the light of the following limitations: First, students from only one city (Krakow) participated in the research, so the study is local. Therefore, it would be desirable to compare the findings with similar studies done in other cities, and to look at this issue in a broader perspective, i.e. in the European or even global one. Second, the findings only represent the opinions of those students who agreed to participate in the study, which is why they cannot be representative of the entire population of students, either in Krakow or in Poland. Third, it should be noted that a fairly large group of students (1605 people) opened the link with the survey questionnaire, but did not complete it. This may indicate the fact that the issue under study involves matters that are difficult, very personal, internal, and sensitive. Fourth, the reasons for using psychoactive substances were not analysed, so it is worth to undertake further and deeper studies in this area. Finally, another limitation is the COVID pandemic which resulted in the study being conducted online rather than in person. This could have affected how much substance use the students reported since now (due to lockdown) social events have been canceled and they spend more time in dorms.

## Conclusions and Implications for Education

As the analysis of the survey findings shows, alcohol is the most popular psychoactive substance used by the students who took part in the study. This is likely due to its availability and the cultural patterns of behavior that Polish people follow at social or family meetings. The students also use marihuana. This is alarming, because this is the substance with which drug initiation usually starts. As for the differences between male and female respondents, women take fewer psychoactive substances, which may reduce the risk of addiction in this group in a long-term perspective. Men, in turn, use more psychoactive substances, which may influence and increase their risk of falling into addiction. Moreover, for a young person, using psychoactive substances is just one of numerous experiences, albeit a very risky one. That is why it is important to minimize the risk factors and create proper conditions for personality development through reinforcing protective factors.

Based on the analysis of the findings, we can conclude that the results of statistical tests make it possible to verify and accept the hypothesis about the correlation between the students’ spirituality and their use of selected psychoactive substances. The higher the level of spirituality (the protective factor), the lower the amount of psychoactive substances the students take. Therefore, we can assume that spirituality is a protective factor. We should develop and reinforce protective factors against use of psychoactive substances, which will consequently reduce the risk factors.

In a world full of chaos, uncertainty and stress that prompt young people to use psychoactive substances, a person may experience joy and peace through spiritual development. Spirituality is a very important component of human resources: a factor that protects us against the consequences of abuse and addiction to psychoactive substances.

There is no doubt that spirituality is one of such protective factors. In human life, it performs a meaning-creating, directing, stabilizing, indicating, integrating-complementing, and normative function. The meaning-creating function helps a person discover the meaning of life and give life a meaning. It helps us answer the question: why do I live? The directing function helps us go into a particular direction, realize the meaning of life, and answer the question: where am I going? The stabilizing function gives us a sense of security and satisfies our basic social needs: acceptance, the sense of belonging and relationships. The indicating function shows us the way and means of achieving the goal and meaning of life; it helps us answer the question: where am I? The integrating-complementing function integrates our personality and all dimensions of life, and it makes it possible for us to answer the question: who am I? The normative function points to the principles and norms which we should follow in order to be happy (Borda & Solecki, 2017).

The findings clearly indicate that there is a correlation between young people’s use of psychoactive substances and their level of spirituality. People with a higher level of spiritual development use less psychoactive substances. That is why it is worth taking into account the spiritual sphere in education. This should also take place with regard to students. The period of studies cannot be limited to biological-physical, cognitive-intellectual, psychological-volitional, and relational-social development. It should also be mindful of the transcendental-spiritual growth.

Since the causes of using psychoactive substances have not been analyzed, it is worth continuing the research in this area. It would also be valuable and interesting to carry out comparative studies showing the issue of spirituality and its relationship to use of psychoactive substances by students not only in Poland but also in Europe or other parts of the world.

## Footnotes

1 The use of the ASP questionnaire in the empirical research was approved by the authors of the questionnaire.

2 Survio is a tool for making online surveys and it is available at https://www.survio.com/pl/ (accessed: 10.11.2021).
